# Lessons from Smart Cart 2.0: Considerations for Integrating Purchasing Data into Healthy Eating Interventions with AI Approaches^☆^

**DOI:** 10.1016/j.tjnut.2026.101604

**Published:** 2026-05-20

**Authors:** Maya K Vadiveloo, Alison Tovar, Anne N Thorndike

**Affiliations:** 1 Department of Nutrition, University of Rhode Island, Fogarty Hall, Kingston, RI, United States; 2 Department of Behavioral and Social Sciences, School of Public Health, Brown University, Providence, RI, United States; 3 Department of Medicine, Division of General Internal Medicine, Massachusetts General Hospital, Boston, MA, United States; 4 Harvard Medical School, Boston, MA, United States

**Keywords:** food purchasing, artificial intelligence, machine learning, digitalized food environments, personalized dietary interventions

## Abstract

The increasing prevalence of chronic diseases related to suboptimal diet quality in the United States and worldwide contributes to burgeoning healthcare costs and excess death and disability. Changing dietary intake is essential but difficult because dietary behaviors stem from interconnected biological, psychological, environmental, and social factors that are rarely comprehensively addressed in interventions. Emerging digital technologies present a paradox in food environments: some facilitate healthier eating, whereas other practices, like targeted marketing and price manipulation, exploit behavioral vulnerabilities and promote poor dietary habits. Smart Cart 2.0—a personalized digital healthy eating intervention using artificial intelligence and machine learning to detect patterns and influence dietary choices—offers critical lessons for health technology design. This perspective contends that improving digital dietary behavior interventions hinges on responsible implementation: fully leveraging technology’s promise while establishing safeguards against premature adoption. With appropriate guardrails, digital technologies provide a tremendous opportunity to improve the delivery, personalization, and scalability of healthy eating interventions.

## Introduction

Improving the design and delivery of healthy eating interventions is critical to reducing the burden of diet-related chronic disease. One promising avenue for enhancing these interventions involves personalized digital technologies. Although these technologies cannot deliver personalized dietary guidance without human oversight, their rapid improvement offers promise for scalable individualized interventions.

One compelling area is the digitalized food environment, where technology is rapidly transforming how individuals access, purchase, and consume food, offering new opportunities to improve diet quality while raising important questions about equitable access, targeted marketing, information accuracy, and data privacy [1]. This transformation is particularly evident in online grocery shopping, where artificial intelligence (AI) and machine learning (ML) applications are reshaping nutrition interventions. This perspective examines considerations for developing AI-enabled tools in digitalized food environments, drawing on lessons from developing Smart Cart 2.0 [2], a personalized digital healthy eating intervention that uses automated grocery receipt analysis and tailored incentives, to improve diet quality.

The Smart Cart 2.0 platform was developed to provide a scalable solution to improve diet quality while addressing multiple determinants of food choice. Established frameworks like the socioecological framework and the 5 A’s (i.e., availability, accessibility, affordability, accommodation, and acceptability) depict how food environments influence diet [3,4], yet interventions rarely address evolving, contextual factors. For example, an appropriately timed health or social cue may override taste and price as the primary drivers of food choice in some situations [5,6]. Although existing healthy eating interventions target determinants like price and availability [7,8], digital technologies are rapidly reshaping this landscape in real time by using advanced analytic tools to promote unhealthy food choices [9].

Food marketers are leveraging AI to shape food choice in unprecedented ways. ML—a subset of AI—learns from large, evolving datasets to generate increasingly accurate predictions over time. This capability is fueled by widespread adoption of digital technologies (i.e., smart devices, phones, tablets, and computers), which generate digital footprints that reveal key factors shaping individual food choice. Food marketers dynamically analyze digital data, including sales data, social media activity, and loyalty program data, to understand consumer behavior and better tailor their products and marketing campaigns to optimize return on investment [10,11]. Meanwhile, public health researchers frequently rely on static, constrained datasets and do not dynamically adapt to evolving data streams [12,13]. Insufficient integration of time-varying dietary behavior data with other health data limits opportunities to provide real-time, personalized guidance that improves adherence to a healthy dietary pattern [11,14]. The lack of integration between dietary data (e.g., food pricing, neighborhood food access) and health data (e.g., electronic health record data) continues to limit the scalability of long-term personalized behavior change interventions that rely on strategies like priming and nudges [15,16]. Smart Cart 2.0 was designed to address these limitations by bridging this data integration gap through advances in digital technology.

Developing Smart Cart 2.0 has revealed 4 critical considerations for AI-enabled nutrition tools that warrant broader discussion, including how they: *1*) facilitate development of dynamic, responsive interventions that adapt to individual behavior patterns; *2*) create opportunities and barriers for equitable access, particularly within online retail spaces; *3*) raise questions about the appropriate balance between automation and expert guidance; and *4*) motivate discussions about applying ethical frameworks to protect privacy when integrating dietary and health data. These considerations are not intended to be one-size-fits-all, but rather to describe aspects revealed during the formative design that could be applicable to others developing AI-informed dietary tools. Although AI applications in nutrition span clinical care, public health surveillance, food systems, and policy, this article focuses on digitalized food retail because online shopping has fundamentally restructured the food environment and created new opportunities to analyze and influence food choice determinants [1]. These considerations have implications for researchers, clinicians, and policymakers seeking to harness AI for nutrition interventions while minimizing potential harms.

### The smart cart 2.0 case

In 2018, our research team began to explore whether human expertise combined with AI/ML could be used to integrate dietary preferences with continuous grocery receipt data to deliver personalized coupons for healthier food choices. In a 9-mo randomized clinical crossover trial (Smart Cart) [17], when participants received personalized, weekly healthy food coupons linked directly to their supermarket loyalty card, they spent more on healthy foods and improved the healthfulness of their grocery purchases. The study’s semiautomated algorithm used the loyalty card-linked receipt data to select personalized healthy food coupons that aligned with participant-specified dietary preferences and could improve their grocery purchase quality.

The preliminary success of this intervention led to current efforts to refine the platform (Smart Cart 2.0) to make it fully automated and to convert it from a weekly e-mail with coupons usable at a single retailer to a web application that can be used across multiple online retailers [2]. These refinements enable Smart Cart 2.0 to respond to dynamic dietary behaviors, expand healthy food access through online grocery integration, and provide the infrastructure needed to scale.

**FIGURE 1:**
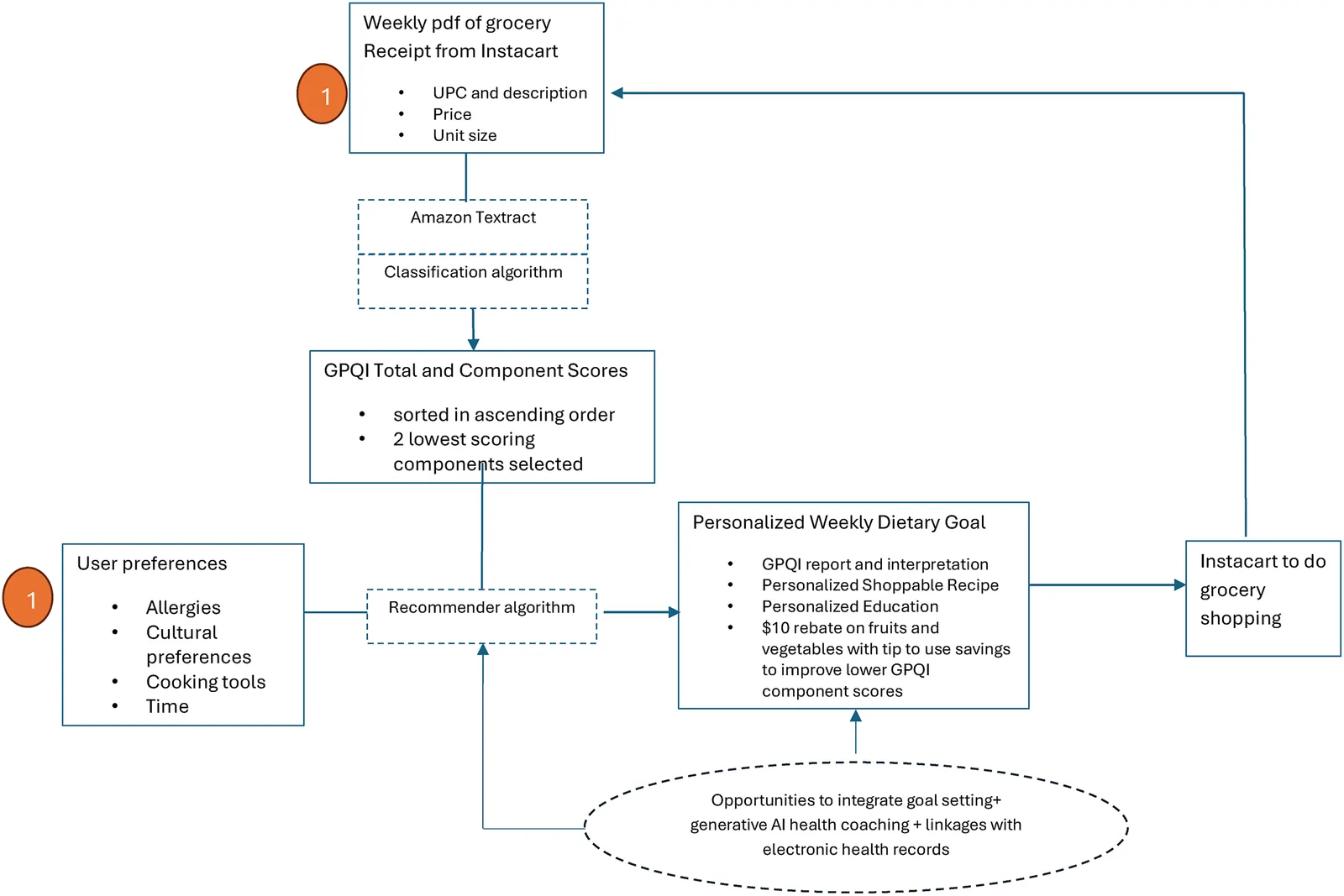
Applications of machine learning to improve the design and delivery of healthy eating interventions: a case example from the Smart Cart 2.0 Study. The logical flow of the Smart Cart 2.0 platform is shown. Smart Cart 2.0 requires 2 user inputs (identified using 2 circles with the number 1) to generate GPQI (Grocery Purchase Quality Index) Total and Component Scores and Personalized Dietary Goals. The conceptual diagram of the platform, including areas where advanced applications of AI could be incorporated, demonstrate how digitalized food environments could capture relevant behavioral data, integrate with other health data, and improve the delivery of personalized healthy eating interventions. As depicted, AI tools are being used to extract receipt data using Amazon Textract, to classify that data using natural language processing into GPQI categories, to recommend personalized recipes, and to make the recipes shoppable. AI, artificial intelligence; GPQI, Grocery Purchase Quality Index; UPC, Universal Product Code.

Briefly, Smart Cart 2.0 uses a natural language processing-based classification algorithm to identify foods on a grocery receipt and calculate the Grocery Purchase Quality Index (GPQI) total and subcomponent scores [18]. A shopper’s GPQI score is strongly correlated with the Healthy Eating Index and reflects the healthfulness or quality of their purchases across 11 adequacy (i.e., total fruit, whole fruit, vegetables, greens and beans, whole grains, dairy, total protein foods, seafood, and nuts) and moderation (i.e., refined grains, processed meats, sweets) subcomponents. To do this, we developed a classification algorithm utilizing data from the original Smart Cart Study purchasing database and publicly available data from the Open Grocery Database [19]. The classification algorithm uses natural language processing to identify key terms from the product’s text description on the receipt to classify it into a food group using a logistic regression package in Python. We used 75% of the data for training and 25% for testing and achieved a weighted mean accuracy of 88% for the 29,000 Universal Product Codes (UPCs) coded to date. When new products are detected on a receipt, the algorithm provides a probabilistic GPQI match, and a research assistant confirms the classification before it is added to the product database. Subsequently, a recommender algorithm prioritizes the 2 lowest-scoring GPQI subcomponents as “areas of improvement,” and combines those areas with the user’s prespecified preferences to identify a relevant recipe, education tip, and nutrition incentive designed to help the user purchase foods that would improve their GPQI score. The backend of the webapp relies on a relational database of tagged recipes and education content curated by the Smart Cart team. Nutrition students identified existing recipes and nutrition education from the Mayo Clinic [20] and other reputable websites [21] that adhered to Dietary Approaches to Stop Hypertension guidelines [22]. Recipes were all coded to determine how well they aligned with different areas of improvement and participant preferences. For example, a recipe for stir-fried shrimp with brown rice and vegetables would improve whole grain, vegetables, seafood, and nuts GPQI subcomponents. The algorithm might match this recipe to an omnivore with celiac disease and stove access with a low score on relevant GPQI subcomponents while excluding it for an individual following a vegetarian pattern. Informed by qualitative feedback [2], the recipe is “shoppable,” with individuals able to directly add recipe ingredients to their cart and select the grocery store where they want their order fulfilled, which helps keep meal planning and shopping in one place. This connection is enabled through an Application Program Interface (API), which allows the Smart Cart 2.0 server to connect directly to the online grocery platform. The proprietary API pulls the ingredients in a recipe and searches the ingredient name in the items list [23]. Product match accuracy is enhanced when recipes conform to the LineItem format, but users are advised to check the accuracy of added ingredients. Users can dynamically alter dietary, cooking, and education tip preferences in Smart Cart 2.0, which will change recipe recommendations and education content. This functionality reduces the cognitive burden associated with finding recipes that align with varied preferences and health goals. The logical flow of the Smart Cart 2.0 platform is presented in Figure 1.

Smart Cart 2.0 represents a responsive intervention that can adapt and learn from dynamic data to better personalize dietary guidance and facilitate healthier food purchasing. Nevertheless, efforts to scale must explicitly consider contexts in which human or expert oversight is critical to mitigate inequities in access and reduce risk of misuse. This was evident when potential Smart Cart 2.0 users [2] reported spending more than intended at times when using online shopping if a sale price could not be honored because of limited stock and inappropriate substitutions. For example, yogurts priced at 5 for $10 would be charged at the higher per-container price if the store only had 4 containers in stock, without alerting the person to the price change beforehand. These additional and unanticipated charges adversely impact food security for some families. Further, some consumers reported price differences between online and in-person shopping that extended beyond taxes and delivery fees, suggesting that families must carefully weigh trade-offs among transportation costs, fuel expenses, product pricing, and time when deciding where to shop.

Some AI-enabled features requested by users, like goal setting, health coaching, or linkages to other health data, currently require expert oversight to integrate [2]. Linking dietary and health data, applying ML to make inferences about data patterns, and applying generative AI tools to tailor healthy eating advice to users requires sophisticated data protection. The decision to limit initial AI features and integration with health data in Smart Cart 2.0 was deliberate, as the current risks of deploying these tools outweigh the potential benefit. As concerns about AI grow, it also provides adequate time to evaluate the optimal ratio of human to AI interactions in long-term healthy eating interventions. Although AI can synthesize dynamic information from varied data sources and provide responsive guidance, biases remain, and it is unclear whether users equally trust AI-based guidance compared with clinician-based guidance [24]. Similarly, as AI performance exceeds expert performance in some domains [25], determining whether AI-driven tools adequately foster patient accountability for health behavior change and who is liable for patient outcomes remains underexplored [24,26]. This work highlights some foundational considerations for researchers to consider as AI applications in nutrition undergo rapid evolution and expansion.

Consideration 1: AI-enabled nutrition tools can create adaptive dietary interventions by leveraging purchasing data.

Development of the Smart Cart 2.0 platform reinforced the benefits of applying AI-enabled nutrition tools in the design and delivery of scalable healthy eating interventions. Dietary behaviors are inherently dynamic, varying across days, months, and seasons, and shaped by a complex interplay of food-related (e.g., price, availability), individual (e.g., taste and health preferences), and societal factors (e.g., food policies, social networks) [11,27]. Public health researchers and food marketers have traditionally relied on focus groups and surveys to uncover preferences. Now, digital data—especially sales records—enable faster, more accurate, and cost-effective ways to detect dietary patterns. It also can provide a dynamic representation of diet, enabling guidance that adapts to contextual factors and shifting preferences in real time.

Although imperfect, one promising area for ongoing development of AI-enabled nutrition tools lies in the use of routinely collected purchasing data (e.g., supermarket or cafeteria sales), which provides a rich source of information about individual preferences. Purchasing data can monitor diet quality at the population level and serve as a foundational input for digitally enabled interventions to shift population-level eating behaviors. The validity of using purchasing data as a proxy for individual dietary data became more common after the Food Acquisition and Purchase Study conducted by the United States Department of Agriculture [28] and the development and validation of the GPQI [18], which demonstrated a valid method for translating the limited information on a grocery receipt into a diet quality score that was reflective of individual diet quality [29]. This index, alongside other advancements that have enabled purchasing data to monitor population dietary quality [30], has been instrumental in creating infrastructure for dynamic, digitally enabled interventions like Smart Cart 2.0. Moreover, it provides the research community with an opportunity to strengthen dietary assessment methods and address existing weaknesses in the validity and reliability of self-reported dietary data as a measure of usual intake [31].

Consideration 2: Online shopping and other digital tools can improve equity in access to healthy eating interventions with appropriate safeguards.

Although most adults across the income spectrum used digital tools to interact with Smart Cart 2.0, other barriers may affect equitable access to healthy eating interventions. A common criticism of technology-leveraged interventions is that individuals may have differential access to smart devices and have barriers related to technological and financial literacy [32]. Although such concerns are not unfounded, recent data demonstrate the diffusion of many forms of technology across all segments of the population. Pew Research Center reports >90% smartphone ownership in the United States in 2024 [33]. Online grocery shopping is also growing across all United States households, and although prevalence is highest among younger individuals (aged 15–24 y) [34], a 2024 report from the Food Industry Association estimates that 67% of consumers do some grocery shopping online [35]. Moreover, both Supplemental Nutrition Assistance Program (SNAP) and non-SNAP recipients largely have favorable opinions of online grocery shopping, finding it easy to use and time-saving [36]. Such positive perceptions are important given that the lack of access to full-service grocery stores is a barrier to healthful dietary patterns in some populations [37]. Nonetheless, logistical and financial barriers, including delivery fees and minimum purchasing amounts, can be difficult to surmount, as can the requirement of having a bank account and backup method for purchase.

Digitalized food environments are not a panacea for addressing equitable access to healthful foods for other reasons as well. As online shopping and self-service kiosks replace human cashiers, unique problems for lower-income and technologically illiterate populations may develop. For example, access to certain digital coupons is currently limited to those with smartphone access. Although smartphone ubiquity is increasing, the most underresourced populations (i.e., very low-income and older adults) may lack access or technological savvy to utilize digital coupons [38]. Concerns about the digital divide and sophisticated use of smart technology were raised during key informant and user interviews in the first phase of Smart Cart 2.0 development [2]. Although the technological literacy to use Smart Cart 2.0 was deemed acceptable, we were cautioned not to integrate too many more features and to consider including a brief handout for users who may need additional support. Other best practices for ensuring ethical technology integration include the use of opt-in/opt-out features to provide more choice to users about where they want technology integration, the use of age-appropriate designs, engaging users in codesign, and ensuring that all data privacy practices are transparent and clearly communicated to users [39].

The mixed impact of the broader digital retail environment on dietary behaviors and diet quality has led to calls to reconceptualize the 5 A’s of the food environment [1]. For example, food access has traditionally been assessed using measures such as proximity to grocery stores and the availability of transportation to reach them. Food environment digitalization necessitates updating this measurement to capture how food access has both expanded and restricted access to different retail options. Although digitalization has reduced some geographic restrictions and transportation requirements, it has introduced new barriers like varied offerings, pricing, and marketing in-person compared with online. Consumers report seeking online shopping for convenience, to expand access to healthy foods, and to reduce impulse purchases. However, various elements of the online grocery experience are beginning to replicate existing retail practices that undermine healthy eating goals [40]. For example, targeted online promotions for unhealthy foods and saved purchase histories can adversely affect the diet quality of consumer purchases [36]. Smart Cart 2.0’s shoppable recipes are free of promotions and purchase histories. However, once users leave the Smart Cart platform, supermarket personalized promotions and “buy it again” suggestions might appear, which could impede healthy eating goals. Further, potent advancements in customized marketing can be used to develop new preferences rather than align with existing preferences [41]. As such, it is important to develop appropriate safeguards to counteract commonly employed marketing strategies to maximize the potential of digital retail to increase healthy food access. Consumer protection oversight and regulation regarding data privacy are necessary to prevent misuse of readily available, limitless data that can be analyzed in real time to craft effective targeted campaigns that shape lifelong preferences [42].

It is within the context of these barriers that innovation within personalized nutrition initiatives may be most important. Unlike brick-and-mortar stores, where shoppers have ingrained pre-existing patterns of behavior, healthy eating interventions within online grocery stores provide opportunities for innovation that could benefit all groups of shoppers. As the population continues to adopt online modalities to buy groceries, it is increasingly compelling to incorporate features like automatic nutrition incentives, goal setting, and meal planning in a normative manner.

Consideration 3: Personalized digitally enabled healthy eating interventions must supplement rather than replace expert guidance.

The integration of AI tools into Smart Cart 2.0 revealed some limitations that require human oversight to mitigate. For example, some errors exist in the product matching feature in emerging “shoppable recipe” tools, which enable all ingredients from a recipe to be immediately added to an individual’s shopping cart and modified as needed. Although this feature is time-saving and helpful to consumers, it can make errors and select inappropriate items or amounts, which need human editing (e.g., a recipe with the ingredient “whole wheat pasta” might lead to a match of wheat germ because the term “pasta” is insufficiently specific; similarly a recipe that uses 1 tablespoon of raisins may match with small boxes of raisins rather than a larger, more economical container). As a result, the consumer must make sure to read and understand a recipe’s basic steps rather than indiscriminately add ingredients to their cart. Although the extent of errors in the “shoppable recipe” feature is currently unknown and likely to change, our preliminary testing suggests that it will be important to communicate with users about the need to carefully review shopping lists.

Nevertheless, extensive use of smart devices and digital technology, combined with processing speed and growth in the accuracy and relevance of AI-enabled nutrition tools, could lead to a paradigm shift in the creation of scalable healthy eating interventions [43]. Increasingly sophisticated AI- and ML-based systems capable of simulating human analytical reasoning offer a promising pathway to detect trends, predict risk, and provide dietary guidance in response to individual behavior [44]. In some domains, like diabetes management, ML-derived models are increasingly integrating continuous glucose monitoring with food intake and activity logs to generate and nudge individualized dietary recommendations, illustrating how similar approaches could transform dietary guidance more broadly in both clinical and public health settings [45].

Ongoing issues with lack of transparency and reproducibility, risk of bias, and security and privacy concerns should preclude use of these models as stand-alone tools [46]. A scoping review [47] noted that clinical use of ChatGPT has utility in providing psychotherapeutic guidance, diagnosing illness, and engaging in therapeutic conversations with patients, while simultaneously noting concerns about it providing overly pessimistic prognostic outputs. Although AI may integrate more seamlessly into online spaces and provide helpful nudges to promote health behaviors, safeguards are necessary both to ensure information accuracy and prevent misuse of consumer data. A second concern related to overreliance on AI tools relates to emerging evidence about the ways automation is reducing critical thinking and reasoning [48], which, when combined with information inaccuracy, presents considerable risk. AI “hallucinations” or incorrect, misleading, or biased results generated by AI models are increasingly reported. Akin to a person following Global Positioning System (GPS) instructions to a familiar location even if they know the directions are wrong, overreliance on AI for health behavior changes risks could lead to the erroneous adoption of potentially harmful health behaviors. As such, without appropriate expert guidance, healthy eating interventions that leverage AI have the potential to exacerbate disparities by missing relevant contextual information [26,44]. Furthermore, unequal access to education on discerning inaccurate, misleading, or biased recommendations may allow AI systems, if left without oversight, to widen existing socioeconomic disparities.

A 2026 systematic review found that misinformation in generative AI-based nutrition tools primarily stems from poor-quality dietary data or AI hallucinations [46]. To address these issues, researchers are increasingly applying strategies that ensure that large language models (LLMs) cite their data sources when making recommendations, which is achieved by leveraging structured data sources like knowledge graphs (i.e., digital relational databases connecting expert-curated lists like recipes, ingredients, and allergens). Multiagentic systems are improving the reliability of AI tools by enabling LLMs to generate tasks and delegate execution to validated tools—though performance remains inconsistent at scale. Even so, expert oversight remains critical for nutritional evaluation and health-specific recommendations. Misinformation persists, often influenced by how precisely users frame their queries. Without verification, generative AI may draw incorrect inferences—such as assuming gluten-free or lactose-free products are inherently healthier.

Consideration 4: Ethical frameworks that safeguard data privacy are essential for integrating purchasing and dietary data with other health data, which could improve health outcomes.

A subset of Smart Cart 2.0 users believed it would be beneficial to link the platform with other health and fitness apps they used [2]. However, we intentionally avoided directly integrating Smart Cart 2.0 with health data, given the dual opportunities and risks that purchasing and health behavior pose for health outcomes and equity (Supplemental Table 1). Many of the opportunities already highlighted include individualized dietary guidance and enhanced access to healthy foods to reduce disease risk and even healthcare spending. These opportunities may be further personalized and impactful when combined with other health data. There is robust evidence that disease prevention is considerably more cost-effective than disease treatment, and preliminary evidence suggests that incentivizing individuals to track and improve dietary behaviors can elicit positive behavior change [49], which, if sustained, would improve health outcomes. However, it is also apparent that unfettered access to such data currently presents considerable risk [44]. For example, ML-based applications that can quickly analyze purchasing trends are being used to unfairly raise or lower food prices in response to real-time demand. Without adequate consumer protection, this ML application may contribute to nutrition and food insecurity. Similarly, as insurance companies gain access to protected health data alongside purchasing information, it may lead to discriminatory pricing and coverage for individuals identified as engaging in health-harming behaviors [26]. Justifiable fears about health insurance and employment discrimination related to substance use, sexual behavior, and mental health concerns may prevent many people from honestly disclosing health behaviors and concerns to their providers.

Consumer concerns regarding data privacy and security are well-documented, and to date, no clear solutions have emerged. A recent review [50] emphasized how higher perceived privacy risks reduce consumers’ intentions to use personalized food platforms, but little is known about how to enhance consumers’ sense of safety and trust. In the absence of strict data privacy regulation, the use of risk-benefit evaluation models like the privacy calculus theory may help with the more effective design and implementation of personalized food and nutrition platforms. In the current landscape, it is also essential to ensure sensitive medical and demographic data are anonymized and housed on a local, third-party hosted server (compared with cloud), with documented retention controls and governance protocols to adequately safeguard data [46].

Coupled with data safety, privacy, and bias concerns is the need to ensure that digital technologies are thoughtfully integrated with human oversight and expertise. Even with powerful predictive analytics, AI models are not always superior to expert guidance, and AI hallucinations with respect to medical decision-making present tremendous risk. Despite the promise of AI as a transformative force in healthcare, harnessing its potential requires ethical oversight of its use in informed clinical and public health decision-making [26].

In conclusion, with appropriate regulation, digitalized food environments and AI/ML applications hold tremendous potential to advance precision nutrition and expand equitable access to personalized dietary guidance at scale. The evolution of Smart Cart 2.0 has shown that expertise combined with AI/ML is a promising strategy for using purchasing data and selected dietary preferences to deliver a personalized healthy dietary intervention. Although future research must evaluate whether Smart Cart 2.0 improves diet quality and reduces chronic disease, insights from its design may be helpful in informing the development of other digital health technologies. Notably, a digital tool that is dynamic and responsive to contextual factors that influence food choice and facilitate healthy meal planning is appealing to consumers. In addition, limiting integration of sensitive data with other apps and ensuring expert oversight of recommendations is important for fostering trust. Future research priorities, including rigorous real-world testing, robust regulatory oversight and data safety protections, consumer education, and integration with expert guidance, will help ensure that AI tools improve rather than exacerbate entrenched suboptimal diet quality and diet-related chronic diseases.

## Author contributions

The authors’ responsibilities were as follows – MKV: took responsibility for drafting the manuscript; AT, ANT: revised the document critically for important intellectual content; and all authors: edited, read, and approved the final manuscript and have primary responsibility for the final content.

## Declaration of Generative AI and AI-assisted technologies in the writing process

During the preparation of this work, the author(s) used a University of Rhode Island supported generative AI tool called LibreChat in order to improve grammar. After using this tool/service, the author(s) reviewed and edited the content as needed and take(s) full responsibility for the content of the publication.

## Funding

MKV and this research were supported by a K01 Career Development award from the National Heart, Lung, and Blood Institute (5K01HL165104). ANT was supported by NIH K24 grant HL163073. The sponsor had no role in the design, collection, analysis, and interpretation of data or writing of the report and decision to submit the article for publication.

## Conflict of interest

The authors report no conflicts of interest.
