# Supplementary material for: Lessons from Smart Cart 2.0: Considerations for Integrating Purchasing Data into Healthy Eating Interventions with AI Approaches
Source: J Nutr. 2026 May 20;156(7):101604. doi: 10.1016/j.tjnut.2026.101604 (PMC13494169; doi:10.1016/j.tjnut.2026.101604)
Supplement: Supplementary file 1 [file mmc1.docx]

Supplemental Table 1: Future Priorities and Elements to Address alongside Advancement of Digitalized Food Environments and Advanced Applications of Machine-Learning and Artificial Intelligence (AI) in Dietary Behavior Surveillance and Change (26, 44)

| **Future priorities** | **Rationale** | **Elements to Address alongside Advancement of tools** |
| --- | --- | --- |
| Implement and validate studies to evaluate the effectiveness of dynamic dietary monitoring and intervention based on purchasing data | - Utilization of evidence-based digital tools could improve consumers’ dietary behaviors and health outcomes | - Reduce algorithmic biases - Improve information accuracy - Ensure data transparency and protection |
| Increase tracking, monitoring, and evaluation of dietary purchases and multilevel drivers of dietary behavior | - Data surveillance facilitates evaluation of environmental changes that influence dietary behaviors | - Ensure consumer protection regulations and oversight - Develop data access regulations for food marketers |
| Enhance access to digital technologies and education | - Widespread access could provide consumers with personalized dietary coaching and behavior change guidance | - Consumer education and training tools to improve consumer literacy, critical thinking, and safety - Equitable access to evidence-based dietary counseling for all individuals |
| Develop systems to integrate AI-based personalized dietary guidance with human oversight into clinical workflows | - Evidence-based personalized digital tools and generative AI-tools could increase both the efficiency and reach of healthcare providers and facilitate personalized dietary guidance | - Identify areas where AI-based systems may assist clinical decision making nad recommendations - Develop regulatory processes for accountability and addressing errors in AI-based guidance and risk prediction |
